# Contribution of muscle stiffness of the triceps surae to passive ankle joint stiffness in young and older adults

**DOI:** 10.3389/fphys.2022.972755

**Published:** 2022-09-05

**Authors:** Kosuke Hirata, Ryota Akagi

**Affiliations:** ^1^ Faculty of Sport Sciences, Waseda University, Saitama, Japan; ^2^ Graduate School of Engineering and Science, Shibaura Institute of Technology, Saitama, Japan; ^3^ College of Systems Engineering and Science, Shibaura Institute of Technology, Saitama, Japan

**Keywords:** elastography, shear modulus, dorsiflexion, aging, gastrocnemius, soleus

## Abstract

This study aimed to investigate whether triceps surae muscle stiffness is associated with passive ankle joint stiffness in 40 young (21–24 years) and older (62–83 years) males. Using ultrasound shear wave elastography, the shear modulus of each muscle of the triceps surae (the medial [MG], lateral gastrocnemius [LG], and soleus [Sol]) was assessed as muscle stiffness at the ankle neutral position (NP) and 15-degree dorsiflexed position (DF15) with the knee fully extended. Passive ankle joint stiffness at the NP and DF15 was calculated as the gradient of the angle–torque relationship at each joint angle during passive ankle dorsiflexion at 1°∙s^−1^ controlled by using an isokinetic dynamometer. Passive ankle joint stiffness was normalized by the body mass. There was no correlation between the absolute ankle joint stiffness and muscle shear modulus of triceps surae in the young and older groups at the NP (*r* ≤ 0.349, *p* ≥ 0.138). Significant positive correlations between absolute ankle joint stiffness and muscle shear modulus at DF15 were observed for MG and Sol in the young group (*r* ≥ 0.451, *p* ≤ 0.044) but not in the older group. The normalized ankle joint stiffness at the NP was significantly positively correlated with the LG shear modulus in young participants and with the MG and LG shear modulus in older participants (*r* ≥ 0.466 and *p* ≤ 0.039). There were significant positive correlations between the normalized ankle joint stiffness and the muscle shear modulus of the triceps surae at DF15 in young and older participants (*r* ≥ 0.464 and *p* ≤ 0.040), except for the MG shear modulus in older participants (*r* = 0.419 and *p* = 0.066). These results suggest that the material properties of the entire triceps surae, even Sol, which is the most compliant muscle among the triceps surae, affect passive ankle joint stiffness, especially when the triceps surae is lengthened and body size is considered.

## 1 Introduction

Joint flexibility is a vital physical fitness factor for most individuals. For instance, high joint flexibility contributes to better athletic performance, such as in gymnastics (Kumagai et al., 2022) and figure skating (Slater et al., 2016), which require extreme joint range of motion (RoM). Additionally, since a wider joint RoM allows resistance to external force for a longer duration, sufficient joint flexibility is required to protect muscle-tendon unit and/or joint structures from external forces, leading to lower injury risks (Witvrouw et al., 2003; Han et al., 2018). Joint flexibility declines with aging owing to age-related changes in the mechanical properties and the morphology of tissues crossing the joint (Harris, 1969; Hirata et al., 2020a). Decreased joint flexibility may require movement pattern alteration, which may lead to a decline in functional abilities, such as balance and walking. Indeed, a narrower RoM in older adults is associated with poor balance ability (Mecagni et al., 2000). Furthermore, a decline in joint flexibility is associated with an increased risk of falls (Campbell et al., 1989). Hence, understanding the determinant factors of joint flexibility is helpful in improving joint flexibility, leading to improvement of athletic performance, reduction in risk of injuries during sports and/or daily living activities, and improvement of the quality of life of older people.

Two major indices of joint flexibility exist: RoM and joint stiffness. Stiffness is a measure of the force/stress required against a certain deformation. The joint stiffness (Nm∙degree^−1^) can be calculated by dividing the joint torque (Nm) by the change in the joint angle (degree). Muscle stiffness is considered one of the major influential factors for both indices of joint flexibility (i.e., RoM and joint stiffness). Recently, muscle stiffness has been indirectly estimated using ultrasound shear wave elastography (SWE) in many studies (Eby et al., 2013; Bernabei et al., 2020). Ultrasound SWE can remotely induce shear waves within the tissues. Because shear waves propagate faster within stiffer materials, their propagation speed within a tissue is associated with localized tissue stiffness. Assuming tissue density and linear elastic behavior, the shear modulus (µ) (an index of stiffness, expressed in kPa) can be calculated using the shear wave propagation speed (ν) as follows: µ = ρν^2^, where ρ is the tissue density. For instance, using this technique, Miyamoto and Hirata (2019) reported that a higher shear modulus of the hamstring muscle (i.e., stiffer hamstring) correlated with the narrower hip flexion RoM and the higher hip flexion stiffness in young adults. Based on this study (Miyamoto and Hirata, 2019), there are no clear differences in the association between the muscle shear modulus and RoM or joint stiffness among the hamstring muscles. In contrast, among the triceps surae, the shear modulus of the gastrocnemii (the medial gastrocnemius [MG] and lateral gastrocnemius [LG]) was more clearly associated with ankle dorsiflexion RoM than the soleus (Sol) (Miyamoto et al., 2018; Hirata et al., 2020a). This lack of clear association between the ankle dorsiflexion RoM and the Sol stiffness may be because Sol is the most compliant muscle among the triceps surae. In most laboratory experiments, including the aforementioned previous studies (Miyamoto et al., 2018; Hirata et al., 2020a), a joint RoM is determined based on the participants’ sensations, that is, discomfort or pain. Since discomfort or pain sensation strongly influences RoM (Weppler and Magnusson, 2010), mechanical stimuli that provoke the perception of discomfort or pain are crucial to RoM. When a muscle is lengthened to a certain length, higher tension (mechanical stimulus) is applied to a stiffer muscle than to a compliant one. Hence, stiffer muscles are expected to cause pain and limit RoM. Because the MG and LG shear moduli are much stiffer than Sol (Hirata et al., 2016), the contribution of MG and LG stiffness to the ankle dorsiflexion RoM may be greater. On the other hand, regarding joint stiffness, which ignores the influence of sensation, not only the MG and LG stiffness but also the Sol stiffness is expected to contribute to the ankle joint stiffness because it is the largest muscle among the triceps surae (Ward et al., 2009). However, to date, the role of intermuscular differences in muscle stiffness among the triceps surae in passive ankle joint stiffness is unknown.

In older adults, no clear association between the muscle shear modulus of the triceps surae and the ankle dorsiflexion RoM was observed (Hirata et al., 2020a; Nakamura et al., 2021). This may be due to age-related decreases in muscle stiffness (Akagi et al., 2015; Yoshida et al., 2017; Do et al., 2021; Nakagawa et al., 2022). As mentioned above, it may be difficult for relatively compliant muscles to cause pain when lengthened. Additionally, aging-induced muscle atrophy and an increase in collagen cross-linking (Avery and Bailey, 2005) may influence the contribution of muscle and non-muscular structures to pain sensation and joint RoM. However, it seems reasonable that muscle stiffness plays a major role in the joint stiffness of the limbs, even in older adults. Despite this, the association between muscle stiffness and passive joint stiffness in older adults has not yet been explored. Stiff joints in older adults may lead to mechanical constraints in daily living activities. Additionally, a potential link between the MG shear modulus and the fall risk has been suggested in community-dwelling older adults (Kim et al., 2022). Therefore, the factors that influence the joint stiffness and the association between muscle and passive joint stiffness in older adults should be elucidated.

This study aimed to investigate whether triceps surae muscle stiffness is associated with passive ankle joint stiffness in young and older adults. Because joint stiffness is a product of the combination of mechanical resistance of several tissues crossing the joint, the contribution of triceps surae muscle stiffness to ankle joint stiffness may change depending on the ankle joint angle. Considering that plantar flexors become much stiffer and dorsiflexors become much more compliant in the ankle dorsiflexed position, the contribution of the triceps surae to the ankle joint stiffness is expected to increase in the ankle dorsiflexed position. Hence, we also investigated the joint angle specificity of the association between muscle and joint stiffness. We hypothesized that 1) stiffer triceps surae would be associated with higher passive ankle joint stiffness regardless of the age group and 2) the association of triceps surae stiffness with passive ankle joint stiffness would be stronger in the ankle dorsiflexed position than in the ankle neutral position (NP).

## 2 Materials and methods

### 2.1 Participants

To compute the required sample size for a correlation analysis in each young and older adult, a priori power analysis was conducted using the G*Power statistical power analysis software (G*Power 3.1.9.7; Kiel University, Germany). A type 1 error and a statistical power were set at 0.05 and 0.80, respectively. The effect size was assumed to be 0.60 according to a previous study (Miyamoto and Hirata, 2019). The critical sample size was determined as 17. Hence, 20 young men and 20 older males were recruited for this study. The present study was part of a larger cross-sectional study conducted in our laboratory to investigate age-related changes in joint flexibility (Hirata et al., 2020a; Hirata et al., 2020b). Table 1 presents the physical characteristics of the participants. The participants were asked to refrain from strenuous exercise for 24 h prior to the experiment. None of the participants reported any muscle soreness or, orthopedic or neurological disorders at the time of the experiment. All the participants were informed of the purpose and risks of the experiment. Written informed consent to participate in this investigation was obtained from all participants. The experimental procedure was approved by the ethics committee of the Shibaura Institute of Technology and was performed in accordance with the Declaration of Helsinki.

**TABLE 1:** Anthropometric data, passive ankle joint stiffness, and muscle shear modulus of the participants.

	Young		Older		Difference				
	Mean	SD	Mean	SD	Delta	95% CI (Lower)	95% CI (Upper)	*p* value	Cohen’s *d*
Anthropometric data									
Age (yr)	22	1	72	5	-	-	-	-	-
Height (cm)	172	6	167	6	5	1	9	**0.011**	0.845
Body mass (kg)	67	11	69	10	−1	−8	5	0.675	0.134
Absolute ankle joint stiffness									
At neutral position (Nm∙degree^−1^)	0.34	0.10	0.32	0.09	0.02	−0.04	0.08	0.606	0.165
At 15-degree dorsiflexion (Nm∙degree^−1^)	0.93	0.27	1.04	0.33	−0.11	−0.30	0.08	0.248	0.371
Normalized ankle joint stiffness									
At neutral position (Nm∙degree^−1^∙kg^−1^)	0.0050	0.0012	0.0048	0.0014	0.0002	−0.0006	0.0011	0.586	0.174
At 15-degree dorsiflexion (Nm∙degree^−1^∙kg^−1^)	0.0137	0.0031	0.0153	0.0044	−0.0016	−0.0040	0.0009	0.203	0.410
Muscle shear modulus									
MG at neutral position (kPa)	8.3	1.0	7.3	1.4	0.9	0.2	1.7	**0.020**	0.766
MG at 15-degree dorsiflexion (kPa)	20.5	5.1	19.8	6.4	0.7	−3.1	4.4	0.721	0.114
LG at neutral position (kPa)	6.8	1.2	6.0	1.1	0.8	0	1.5	**0.040**	0.671
LG at 15-degree dorsiflexion (kPa)	13.5	3.9	12.5	3.4	0.9	−1.4	3.3	0.425	0.255
Sol at neutral position (kPa)	5.0	0.9	4.5	0.6	0.5	0	1.0	**0.031**	0.707
Sol at 15-degree dorsiflexion (kPa)	7.2	1.5	6.7	1.1	0.5	−0.3	1.3	0.229	0.386

SD, standard deviation; CI, confidence interval; MG, medial gastrocnemius; LG, lateral gastrocnemius; Sol, soleus.

### 2.2 Experimental procedures

Participants lay prone on a dynamometer bed (CON-TREX MJ; Physiomed, Germany). Their hips and knees were fully extended. After a visual check to align the rotational axes of the right ankle and dynamometer, the right foot was fixed to the foot plate of the dynamometer with non-elastic straps. Before the actual measurement, five repetitions of passive ankle dorsiflexion and plantar flexion were performed at 5°s^−1^ between 30 degree of plantar flexion (PF30) and 15-degree dorsiflexion (DF15) (NP was defined as 0°) to familiarize the participants with the passive dorsiflexion motion by the dynamometer and to avoid a conditioning effect on tissue stiffness (Konrad & Tilp, 2014). To evaluate passive ankle joint stiffness, passive ankle dorsiflexion was conducted once at 1°∙s^−1^ from PF30 to the angle at which the participant experienced the onset of pain. This joint angle was defined as the maximal dorsiflexion angle. During the ankle joint movement, the participant was asked to relax as much as possible, and we monitored the electromyographic (EMG) activity of the triceps surae to ascertain muscular relaxation. Immediately after the passive ankle dorsiflexion task, the ankle was returned to the plantar flexed position to avoid a stretching effect on muscle stiffness. Subsequently, a muscle stiffness assessment was conducted for each triceps surae muscle using an ultrasonic SWE (ACUSON S2000; Siemens Medical Solutions, United States). The muscle stiffness assessment was randomized and counterbalanced among the participants. For each muscle, muscle stiffness was assessed firstly at the NP and secondly at DF15 with no rest period. Following the completion of this sequential assessment, a 2-min rest period was provided, with the ankle plantar flexed position. Then, the next muscle stiffness assessment was performed. This measurement-rest cycle was repeated until the finishing muscle stiffness assessment for the entire triceps surae was completed. Finally, maximal voluntary isometric contraction (MVIC) of the plantar flexors was performed for 3 s in the NP to normalize the EMG activity. The ankle joint angle, passive joint torque, and the EMG data were simultaneously stored at 1 kHz on a personal computer through a 16-bit analog-to-digital converter (PowerLab 16/35; ADInstrument, Australia).

### 2.3 Muscle stiffness measurement

**FIGURE 1:**
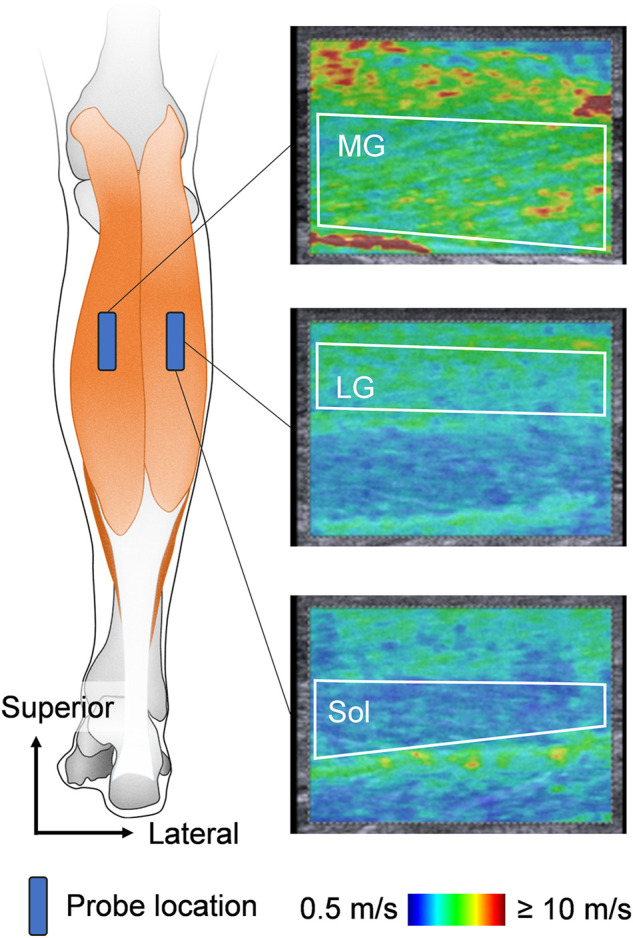
Schematic representation of ultrasound probe locations and typical examples of ultrasound shear wave elastographic images. The area surrounded by the white line is the region of interest for shear wave speed analysis. MG: medial gastrocnemius, LG: lateral gastrocnemius, and Sol: soleus.

In this study, the muscle shear modulus, which was evaluated by SWE using an ultrasonographic device (ACUSON S2000; Siemens Medical Solutions, United States) coupled with a linear transducer array (9L4 Transducer, 4–9 MHz; Siemens Medical Solutions, United States), was used as an index of the muscle stiffness (Figure 1). According to the previous studies (Akagi et al., 2015; Hirata et al., 2020a), the measurement site for the muscle shear modulus was at 30% of the lower leg length from the lateral aspect of the knee joint space to the lateral malleolus. The transverse location of the probe was at the midpoint of the MG width for the MG stiffness measurement and at the midpoint of the LG width for the LG and Sol stiffness measurements. When quantifying the muscle shear modulus, the ultrasound probe was carefully aligned along the fascicle direction of the target muscle using sufficient ultrasound gel. Because the ultrasonic apparatus used in this study cannot evaluate the shear modulus successively, one elastographic image was obtained for each muscle at each joint angle to avoid muscle stiffness reduction owing to the long acquisition time for shear modulus. Additionally, the muscle shear modulus was assessed as quickly as possible to minimize any reduction in muscle stiffness, especially in the measurement at DF15. This procedure was confirmed in a previous study (Hirata et al., 2020a). The quality of the muscle shear modulus measurement was checked using the ultrasound device before storing the image. Muscle shear modulus measurements and analyses were conducted by the same investigator (KH). After the experiment, an additional SWE measurement was performed for four young and older participants to ensure repeatability of the SWE measurement. The coefficient variations of the two measured values of shear modulus for each muscle at each joint angle and the intraclass correlation coefficients for each muscle. The coefficient variations and the intraclass correlation coefficients were 0.47–1.84% and 0.998 to 0.999 for young participants, and 0.32–1.95% and 0.995 for older participants, respectively.

### 2.4 Electromyograph settings

To ascertain muscular relaxation during passive ankle dorsiflexion motion (joint stiffness measurement) and muscle shear modulus measurement, the EMG activity was recorded from the MG, LG, and Sol. Prior to electrode attachment, the skin was prepared (shaving, rubbing with sandpaper, and cleaning with alcohol). Pre-amplified surface EMG electrodes (electrode shape, parallel bar; electrode size, 1 mm × 10 mm; interelectrode distance, 10 mm; DE-2.1, Delsys Inc.) with band-pass filtering between 20 and 450 Hz (Bagnoli-8 EMG System; Delsys Inc., Boston, MA, United States) were then placed. The electrodes were located just beside the muscle shear modulus measurement location for the MG and LG, and midway between the distal myotendinous junction of the LG and Sol for Sol. The reference electrode was attached to the left medial malleolus.

### 2.5 Data analyses

The passive ankle joint stiffness was evaluated using a fourth-order polynomial equation fitted to the passive torque-joint angle curve for each participant (y = ax^4^ + bx^3^ + cx^2^ + dx + e, where y is the torque, x is the ankle joint angle [the NP: 0, positive value: dorsiflexion, and negative value: plantar flexion], and a–e are constants) according to previous studies (Nordez et al., 2006; Herda et al., 2011). The fitting of equation was good (R^2^ = 0.977 ± 0.048 [mean ± standard deviation], 95% confidence interval of R^2^: 0.961–0.992). The passive ankle joint stiffness (slope of the passive torque-joint angle curve) was calculated at NP and DF15 using the first derivative of the equation (dy/dx = 4ax^3^ + 3bx^2^ + 2cx + d, where dy/dx is the passive joint stiffness). The passive ankle joint stiffness was normalized by the body mass.

The procedure for analyzing the muscle shear modulus assessed by ultrasound elastography has been previously described in detail (Hirata et al., 2020a). Briefly, elastographic images were exported from the ultrasonic device in DICOM format. A target muscle was extracted from the color-coded area of the image as much as possible while excluding non-target tissues using an image processing software (ImageJ; NIH, United States). The shear wave speed of each pixel was computed using our original analysis software written in MATLAB (MATLAB R2018a; MathWorks, United States), which converts the RGB values of each pixel into shear wave speed values according to the color scale of the elastographic image. The shear wave speed (ν) was converted to the shear modulus (µ) for each pixel using the formula µ = ρν^2^, where ρ is the muscle density (1.084 g/cm^3^) (Ward and Lieber, 2005). The mean value of the shear modulus over the region of interest of each image was calculated as the muscle stiffness.

For the EMG data, the root mean square (EMG-RMS) value for each muscle was calculated over the entire period of passive ankle dorsiflexion motion (passive joint stiffness measurement) and a 500-ms period at NP and DF15 during the muscle stiffness measurements. The EMG-RMS values were normalized to the EMG-RMS values for 500 ms during MVIC.

### 2.6 Statistical analyses

Normality of distribution was checked using the Shapiro–Wilk test. An independent *t*-test was performed to compare the physical characteristics, passive ankle joint stiffness, and muscle shear modulus between young and older participants. As the EMG activity was not normally distributed, the Mann–Whitney U test was conducted to compare young and older participants. A Pearson product-moment correlation analysis was conducted to investigate the association between the absolute and normalized ankle joint stiffness and the muscle shear modulus. The significance level was set at *α* = 0.05. Data are presented as mean ± standard deviation unless otherwise indicated. A 95% confidence interval for the difference or correlation coefficient was reported. Effect sizes were also reported as follows: Cohen’s *d* for the independent *t*-test, *r* for the Mann–Whitney U test, and *r* for correlation analysis. We considered the effect sizes for the independent *t*-test *d* ≥ 0.8 as large effects, ≥ 0.5 as medium effects, and ≥ 0.2 as small effects; effect sizes for the Mann–Whitney U test *r* ≥ 0.5 as large effects, ≥ 0.3 as medium effects, and ≥ 0.1 as small effects; and effect sizes for correlation analysis *r* ≥ 0.5 as large effects, ≥ 0.3 as medium effects, and ≥ 0.1 as small effects (Cohen, 1988). All statistical analyses were performed using statistical software (SPSS Statistics 28.0; IBM Japan, Japan).

## 3 Results

### 3.1 Age-related differences in passive joint stiffness, muscle shear modulus, and electromyograph activity

Table 1 shows the absolute and normalized ankle joint stiffness and the muscle shear modulus in young and older participants. There was no significant difference in passive joint stiffness between young and older participants (*p* ≥ 0.203, *d* ≤ 0.410). Muscle shear modulus of the triceps surae in the young group was significantly higher at the NP than that in the older group, with moderate effects (12–13%; *p* ≤ 0.040, *d* ≥ 0.671). For the muscle shear modulus of the triceps surae at DF15, no significant difference was observed between young and older participants (*p* ≥ 0.229, *d* ≤ 0.386). Table 2 shows the EMG activity of the triceps surae in young and older participants. EMG activity was significantly higher in the older participants than in the young participants, in MG and LG during the passive ankle dorsiflexion with moderate effects (1.1–1.5%MVIC; *p* ≤ 0.046, *r* ≥ 0.448), and MG and LG during the muscle stiffness measurement with large effects (1.1–1.3%MVIC; *p* < 0.001, *r* ≥ 0.726).

**TABLE 2:** Electromyography (EMG) activity of the participants.

	Young			Older			Mann–Whitney U test	
	Mean	Median	IQR	Mean	Median	IQR	*p* value	*r*
EMG activity during passive ankle dorsiflexion								
MG (%MVIC)	1.5	1.2	1.6	2.6	1.8	2.6	**0.021**	0.514
LG (%MVIC)	2.5	1.6	1.8	4.0	2.5	3.0	**0.046**	0.448
Sol (%MVIC)	3.2	2.7	3.7	5.1	2.9	5.8	0.289	0.242
Muscle shear modulus								
MG at neutral position (%MVIC)	0.8	0.5	0.5	1.9	1.5	2.2	**<0.001**	0.729
MG at 15-degree dorsiflexion (%MVIC)	0.8	0.5	0.6	1.9	1.4	2.3	**<0.001**	0.726
LG at neutral position (%MVIC)	1.2	0.9	0.7	2.5	2.3	2.0	**<0.001**	0.798
LG at 15-degree dorsiflexion (%MVIC)	1.2	0.9	0.8	2.5	2.3	2.0	**<0.001**	0.786
Sol at neutral position (%MVIC)	1.4	1.1	1.3	2.0	1.4	1.7	0.165	0.311
Sol at 15-degree dorsiflexion (%MVIC)	1.5	0.9	1.3	2.0	1.3	1.9	0.072	0.405

IQR, interquartile range; MG, medial gastrocnemius; LG, lateral gastrocnemius; Sol, soleus; MVIC, maximal voluntary isometric contraction.

### 3.2 Correlations of absolute joint stiffness with muscle shear modulus

Table 3 shows the correlation coefficients of absolute ankle joint stiffness with the muscle shear modulus of the triceps surae in young and older participants. No significant correlation was observed between the absolute joint stiffness and the muscle shear modulus at the NP in either group (MG: *r* ≤ 0.349 and *p* ≥ 0.138, LG: *r* ≤ 0.284 and *p* ≥ 0.236, Sol: *r* ≤ 0.195 and *p* ≥ 0.416). The absolute joint stiffness at DF15 was significantly positively correlated with the MG and Sol shear modulus with moderate to large effects but not the LG shear modulus in young participants (MG: *r* = 0.451 and *p* = 0.044, LG: *r* = 0.391 and *p* = 0.084, Sol: *r* = 0.508 and *p* = 0.021). There were no significant correlations in older participants (MG: *r* = 0.090 and *p* = 0.704, LG: *r* = 0.196 and *p* = 0.405, Sol: *r* = 0.300 and *p* = 0.201).

**TABLE 3:** Pearson correlation coefficients between the absolute ankle joint stiffness and muscle shear modulus of the triceps surae.

		At neutral position				At 15-degree dorsiflexion			
Group	Muscle	*r*	95% CI (Lower)	95% CI (Upper)	*p* value	*r*	95% CI (Lower)	95% CI (Upper)	*p* value
Young	MG	0.349	−0.111	0.685	0.138	0.451	0.011	0.745	**0.044**
(*n* = 20)	LG	0.284	−0.181	0.645	0.236	0.391	−0.062	0.711	0.084
	Sol	0.195	−0.271	0.587	0.422	0.508	0.085	0.776	**0.021**
Older	MG	0.181	−0.284	0.577	0.422	0.090	−0.367	0.512	0.704
(*n* = 20)	LG	0.208	−0.258	0.596	0.358	0.196	−0.270	0.587	0.405
	Sol	0.184	−0.281	0.579	0.416	0.300	−0.165	0.655	0.201

CI, confidence interval; MG, medial gastrocnemius; LG, lateral gastrocnemius; Sol, soleus.

### 3.3 Correlations of normalized joint stiffness with muscle shear modulus

**FIGURE 2:**
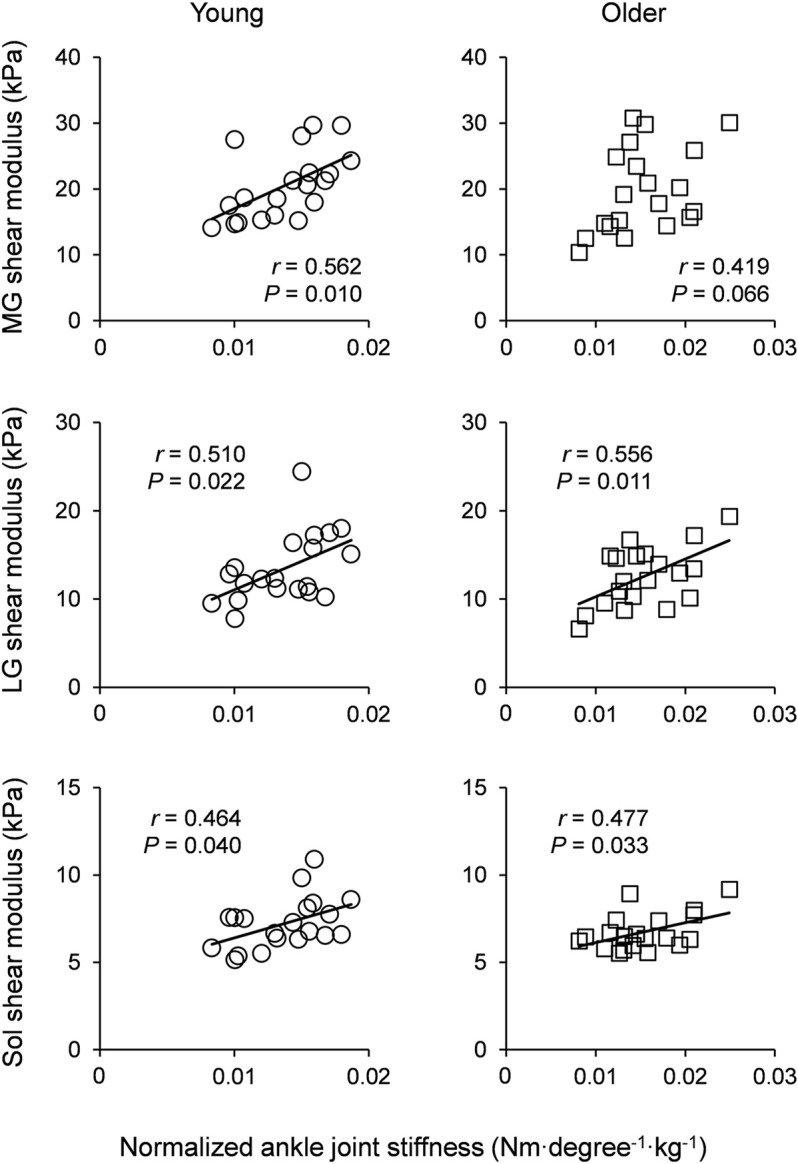
Scatter plots of normalized ankle joint stiffness with muscle shear modulus of the triceps surae for young participants (left column) and older participants (right column). Linear regression lines were drawn for significant correlations as solid lines. MG: medial gastrocnemius, LG: lateral gastrocnemius, and Sol: soleus.

Table 4 shows the correlation coefficients of normalized ankle joint stiffness with the muscle shear modulus of the triceps surae in young and older participants. The normalized joint stiffness at the NP was significantly positively correlated with the LG shear modulus with a moderate effect in the young participants (MG: *r* = 0.424 and *p* = 0.061, LG: *r* = 0.466 and *p* = 0.039, Sol: *r* = 0.280 and *p* = 0.219), and the MG and LG shear modulus with moderate to large effects in the older participants (MG: *r* = 0.483 and *p* = 0.031, LG: *r* = 0.514 and *p* = 0.022, Sol: *r* = 0.204 and *p* = 0.370). There were significant positive correlations between the normalized joint stiffness and the muscle shear modulus of the triceps surae at DF15 with moderate to large effects in young participants (MG: *r* = 0.562 and *p* = 0.010, LG: *r* = 0.510 and *p* = 0.022, Sol: *r* = 0.464 and *p* = 0.040). Moreover, there were significant positive correlations with moderate to large effects in older participants, except for the MG shear modulus (MG: *r* = 0.419 and *p* = 0.066, LG: *r* = 0.556 and *p* = 0.011, Sol: *r* = 0.477 and *p* = 0.033). Figure 2 shows the scatter plots between the normalized ankle joint stiffness and the muscle shear modulus of the triceps surae at DF15 in young and older participants.

**TABLE 4:** Pearson correlation coefficients between the normalized ankle joint stiffness and muscle shear modulus of the triceps surae.

		At neutral position				At 15-degree dorsiflexion			
Group	Muscle	*r*	95% CI (Lower)	95% CI (Upper)	*p* value	*r*	95% CI (Lower)	95% CI (Upper)	*p* value
Young	MG	0.424	−0.023	0.730	0.061	0.562	0.159	0.804	**0.010**
(*n* = 20)	LG	0.466	0.030	0.753	**0.039**	0.510	0.087	0.777	**0.022**
	Sol	0.280	−0.186	0.643	0.219	0.464	0.026	0.752	**0.040**
Older	MG	0.483	0.052	0.763	**0.031**	0.419	−0.029	0.727	0.066
(*n* = 20)	LG	0.514	0.093	0.779	**0.022**	0.556	0.150	0.801	**0.011**
	Sol	0.204	−0.262	0.593	0.370	0.477	0.044	0.759	**0.033**

CI, confidence interval; MG, medial gastrocnemius; LG, lateral gastrocnemius; Sol, soleus.

## 4 Discussion

The main findings of our study were as follows: 1) Absolute ankle joint stiffness at the NP was not associated with any of the muscle shear moduli of the triceps surae in the young and older groups. 2) Absolute ankle joint stiffness at DF15 was associated with the muscle shear modulus in young participants, but not in older participants. 3) Normalized ankle joint stiffness at the NP was associated with muscle shear modulus of the gastrocnemii but not the Sol shear modulus in both groups. 4) Normalized ankle joint stiffness at DF15 was associated with the muscle shear modulus of almost all triceps surae in both the groups. These results partially support our hypothesis. These findings suggest that stiffness of the entire triceps surae, even Sol, which is the most compliant muscle between the triceps surae, affects passive ankle joint stiffness, especially when the triceps surae are lengthened and normalized to body mass.

There was no significant correlation between the absolute ankle joint stiffness and muscle shear modulus of the triceps surae at the NP (Table 3). The lack of correlation between the MG shear modulus and absolute ankle joint stiffness at the NP observed in this study is in line with a previous study (Chino and Takahashi, 2015). In the present and previous studies, the shear modulus was evaluated by ultrasonic elastography as muscle stiffness. The shear modulus (kPa) is a proxy for stiffness and independent of size. Hence, if there are two muscles with the same shear modulus but different sizes, the size-dependent stiffness, such as an index determined as the force required to stretch the unit muscle length (N∙m^−1^), of these muscles is different. Considering that absolute joint stiffness (Nm∙degree^−1^) is a size-dependent measure with individual differences in the body size, it is not surprising that the muscle shear modulus of the triceps surae rarely correlates with the absolute ankle joint stiffness. In addition, the muscle shear modulus develops exponentially with passive muscle stretching (Koo et al., 2014). The muscle shear modulus of the triceps surae at the NP is much less than that at the maximal ankle dorsiflexion (approximately 5–10 times the muscle shear modulus at the NP) (Hirata et al., 2016). Therefore, the absolute ankle joint stiffness is largely influenced by the size of various tissues crossing the joint rather than the material properties of the triceps surae.

While no correlation between the absolute ankle joint stiffness and the muscle shear modulus of the triceps surae was observed at the NP, the absolute ankle joint stiffness at DF15 was associated with the MG and Sol shear moduli in young participants (Table 3). This significant correlation between the MG shear modulus and the absolute ankle joint stiffness in the dorsiflexed position is in line with a previous study (Chino and Takahashi, 2016). MG and Sol are the stiffest (Hirata et al., 2016; Le Sant et al., 2017) and the largest (Ward et al., 2009) muscles within the triceps surae, respectively. Additionally, the MG and Sol at DF15 are much stiffer than those at the NP (Hirata et al., 2016; Le Sant et al., 2017). Hence, the MG and Sol shear moduli can affect the absolute ankle joint stiffness in the dorsiflexed position in young participants, even if the shear modulus is not a size-dependent variable. In contrast, in older participants, there was no correlation between the absolute ankle joint stiffness and the muscle shear modulus of the triceps surae at DF15 (Table 3). Aging decreases muscle mass and changes the quantity and quality of non-muscular tissues, such as thickening of the fascia (Wilke et al., 2019) and increased collagen cross-linking (Avery and Bailey, 2005). This may weaken the contribution of the material properties of the muscle to the absolute joint stiffness. Hence, the muscle shear modulus of the triceps surae may not be associated with absolute ankle joint stiffness in older participants. Collectively, the present results suggest that the association between the muscle shear modulus and the absolute ankle joint stiffness is joint angle, muscle, and age dependent.

Contrary to the absolute joint stiffness, the normalized ankle joint stiffness correlated well with the muscle shear modulus of the triceps surae (Table 4). This may be due to the normalization of body mass. As mentioned previously, the absolute passive joint stiffness is a size-dependent measure. Indeed, a positive correlation between the absolute joint stiffness and the body mass was confirmed in a previous (Takahashi et al., 2018) and in the present study (*r* = 0.384 to 0.471 and *p* = 0.015 to 0.002). Considering these results, the normalized ankle joint stiffness would be more easily associated with the muscle shear modulus than absolute stiffness. Therefore, it is reasonable that the material properties of the triceps surae are associated with the normalized ankle joint stiffness. However, at the NP, the Sol shear modulus did not correlate with the normalized ankle joint stiffness in either group. The Sol is known to be the most compliant muscle in the triceps surae (Hirata et al., 2016; Le Sant et al., 2017) and almost slack at the NP (Hirata et al., 2015; Le Sant et al., 2017). Hence, even if Sol is the largest muscle in the triceps surae (Ward et al., 2009), the material properties of Sol had a negligible impact on the normalized ankle joint stiffness at the NP. On the other hand, MG and LG at the NP were beyond the slack length and moderately stretched in the knee-extended position (Le Sant et al., 2017). These results indicate that only the material property of the gastrocnemii influences normalized ankle joint stiffness at short length of the triceps surae, and the material properties of not only the gastrocnemii but also Sol have impact on the long length of the triceps surae. In particular, this is the first study to reveal that Sol can contribute to passive ankle joint stiffness, that is, joint flexibility, although it has been suggested that Sol has a negligible influence on joint RoM (Miyamoto and Hirata, 2019; Hirata et al., 2020a).

As mentioned and discussed above, a stronger association between the triceps surae shear modulus and the ankle joint stiffness was observed at DF15 than at the NP. These results support our hypotheses. The joint stiffness is a product of the combination of the mechanical resistance of several tissues crossing the joint. Regarding ankle joint stiffness in the sagittal plane movement (i.e., ankle plantar flexion and dorsiflexion), influential factors are not only the plantar flexors, including the triceps surae, but also the dorsiflexors. For instance, in the ankle dorsiflexion position (e.g., DF15), the plantar flexors are stretched and generate high passive tension, whereas the dorsiflexors are slack (Le Sant et al., 2017). Hence, the triceps surae stiffness was strongly associated with the ankle joint stiffness at DF15. At the NP, both the Sol and dorsiflexors are almost slack (Le Sant et al., 2017). In contrast, MG and LG at the NP were beyond the slack length, but much more compliant at DF15 (Le Sant et al., 2017). Therefore, the MG and LG shear modulus (size-independent variable) can only be associated with the normalized ankle joint angle, but the absolute ankle joint stiffness was not correlated with the muscle shear modulus of the entire triceps surae. Regarding older participants, a significant association between the muscle shear modulus and the absolute ankle joint angle was not observed, regardless of the ankle joint angle. This may be because as mentioned above, the material property of muscle has a weaker contribution to the absolute joint stiffness than other tissues, such as the fascia.

The absolute and normalized ankle joint stiffness did not vary between young and older participants, regardless of the ankle joint angle (Table 1). In line with these results, a previous study found no age-related difference in the absolute ankle joint stiffness at a given joint angle (Gajdosik et al., 1999). In the present study, an association between the muscle shear modulus and the absolute ankle joint stiffness was observed in young but not older participants (Table 3). Additionally, as mentioned above, aging may weaken the contribution of muscle and strengthen non-muscular tissues to joint stiffness because aging decreases muscle mass and changes the quantity and quality of non-muscular tissues (Avery and Bailey, 2005; Wilke et al., 2019). These opposite-directed changes in tissue contribution with aging may explain the lack of age-related differences in absolute joint stiffness. Moreover, there was no difference in normalized ankle joint stiffness between the age groups. This is simply because the body mass of young participants was comparable to that of older participants (Table 1).

Joint flexibility is regarded important in numerous aspects such as athletic performance (Slater et al., 2016; Kumagai et al., 2022), sports-related injury risks (Witvrouw et al., 2003; Han et al., 2018), functional ability such as balance and walking (Mecagni et al., 2000), and risk of falls (Campbell et al., 1989). The present findings revealed that the muscle shear modulus of the triceps surae influences the ankle joint stiffness. Hence, modulation of the mechanical properties of the triceps surae (e.g., stretching and warm-up exercises) can be helpful in improving the activities of sports and/or daily living and reducing the risk of injury and/or fall.

This study had several limitations. First, the muscle stiffness was evaluated as the muscle shear modulus using an ultrasound SWE. Although SWE has been widely used to estimate tissue stiffness in recent years, this estimation is based on many assumptions, such as tissue density and linear elastic behavior. In contrast, the validity and usefulness of SWE for muscle stiffness assessments have been reported (Eby et al., 2013; Bernabei et al., 2020). We believe that the muscle shear modulus reflects muscle stiffness. Second, we only assessed the triceps surae. Muscles influencing the ankle joint stiffness are not only the triceps surae, but also other plantar flexor muscles such as the tibialis posterior and flexor digitorum longus. However, the triceps surae occupies approximately 80% mass of the plantar flexors (Ward et al., 2009). Therefore, the muscle shear modulus of the plantar flexors other than the triceps surae is assumed to have a small impact on the ankle joint stiffness. Third, we measured the muscle shear modulus from only one site for each muscle. The muscle shear modulus of the triceps surae is reported to vary intramuscularly (Le Sant et al., 2017). Additionally, we measured the Sol shear modulus at a relatively proximal region, but not where the cross-sectional area of Sol was the largest. However, it is not well understood whether individual or joint angle-dependent differences in the muscle shear modulus vary intramuscularly. We believe that the measurement site of the muscle shear modulus has a negligible effect on the present findings and their interpretation. Fourth, the shear modulus measurements were performed only once for each muscle at each ankle joint angle. Although it may be preferable to assess the shear modulus several times and analyze them for reliability, the repeatability of successive shear modulus measurements has been reported to be extremely high in many previous studies (e.g., ICC ≥ 0.9; Hirata et al., 2020a). Therefore, the assessment of the shear modulus from one elastographic image was not considered problematic. Fifth, slightly higher EMG activity was observed in older participants than in younger participants. Because muscle activity stiffens muscles and joints, the muscle shear modulus and the ankle joint stiffness values shown in this study are not true resting-state values. However, the difference in EMG activity between the age groups was not so large (∼1.5% MVIC), and the EMG activity influenced both the muscle and joint stiffness. Therefore, the observed EMG activities may have a negligible impact on the present findings and their interpretation. Sixth, we measured the muscle and joint stiffness at specific joint angles (the NP and DF15). Since muscle slack length and moment arm is different among individuals, strain of a muscle is not standardized among them in a specific joint angle. When comparing muscle and/or joint stiffness between groups or individuals, it may be necessary to elucidate stiffness-joint angle (muscle length) relationship. Finally, the participants of this study were male. Previous studies have reported sex-related differences in passive joint stiffness (Chino and Takahashi, 2016), muscle shear modulus (Miyamoto and Hirata, 2019), and the association between joint RoM and muscle shear modulus (Miyamoto and Hirata, 2019). Therefore, there may also be sex-related differences in the association between passive joint stiffness and muscle shear modulus. Future studies should clarify this aspect.

## 5 Conclusion

The purpose of this study was to investigate the association between the triceps surae muscle and passive ankle joint stiffness. The results revealed that the muscle shear modulus of the entire triceps surae influences the passive ankle joint stiffness, especially when the triceps surae/ankle joint is lengthened/dorsiflexed and the ankle joint stiffness is normalized to body size. Additionally, even Sol, which is the most compliant muscle between the triceps surae, affects the passive ankle joint stiffness. Moreover, the results also indicate that the association between the muscle shear modulus and ankle joint stiffness is independent of age, although the association is clearer in young than in older adults due to age-related decrease in the contribution of muscle stiffness to joint stiffness. These findings suggest that modulation of the triceps surae stiffness may change the ankle joint stiffness, leading to the improvement of functional ability, such as postural balance and walking, and decrease in injury and fall risks.

## Data Availability

The raw data supporting the conclusions of this article will be made available by the authors, without undue reservation.
